# Prevalence and Associated Factors of Metabolic Syndrome in Patients With Systemic Lupus Erythematosus: A Cross-Sectional Study

**DOI:** 10.7759/cureus.113828

**Published:** 2026-08-02

**Authors:** Shehla Gul, Haider Imran, Roheela Gul, Zabih Ullah, Iffat Siddiq, Juneda Sarfraz

**Affiliations:** 1 Medicine, Kettering General Hospital, Kettering, GBR; 2 Internal Medicine, Khyber Teaching Hospital, Peshawar, PAK; 3 Internal Medicine, Foundation University Medical College, Islamabad, PAK; 4 Medicine, Midland Regional Hospital, Portlaoise, GBR; 5 Medicine, Saidu Group of Teaching Hospitals, Saidu Sharif, PAK; 6 Public Health, Shaheed Zulfiqar Ali Bhutto Medical University, Islamabad, PAK

**Keywords:** cardiovascular risk, metabolic syndrome, ncep, predictors, prevalence, systemic lupus erythematosus

## Abstract

Background: Significant cardiovascular morbidity is associated with systemic lupus erythematosus (SLE), which is also associated with the development of metabolic syndrome (MetS).

Objective: This study aimed to determine the prevalence of MetS and identify its associated factors among patients with SLE.

Methodology: This hospital-based cross-sectional study was conducted from January 2024 to December 2025. A total of 427 adults diagnosed with SLE according to the 2019 EULAR/ACR classification criteria were recruited using consecutive sampling. Demographic, clinical, biochemical, and disease-related data were collected and analyzed using IBM SPSS Statistics for Windows, Version 25.0 (Released 2017; IBM Corp., Armonk, NY, USA). Categorical variables were compared using the chi-square test, continuous variables using the independent-samples t-test, and multivariable logistic regression was performed to identify factors independently associated with MetS.

Results: Among the 427 patients with SLE, 39.34% met the diagnostic criteria for MetS. The most frequent MetS components were low high-density lipoprotein (HDL) cholesterol, central obesity, hypertension, hypertriglyceridemia, and elevated fasting plasma glucose. In the multivariable logistic regression analysis, older age, obesity (BMI ≥ 30 kg/m²), longer disease duration, lupus nephritis, physical inactivity (defined as <150 minutes of moderate-intensity physical activity per week), a family history of diabetes mellitus, and corticosteroid use were independently associated with higher odds of MetS, whereas hydroxychloroquine use was independently associated with lower odds of MetS.

Conclusion: MetS was common among patients with SLE and was independently associated with several demographic, disease-related, treatment-related, and lifestyle factors. These findings underscore the importance of routine metabolic screening and comprehensive assessment of metabolic abnormalities as part of the clinical management of patients with SLE. Given the cross-sectional design of the study, the observed associations should not be interpreted as causal, and prospective longitudinal studies are warranted to clarify temporal relationships and validate these findings.

## Introduction

The chronic autoimmune disease known as systemic lupus erythematosus (SLE) affects multiple organ systems and follows a relapsing-remitting course [1]. It primarily affects women of reproductive age and is associated with significant morbidity due to persistent inflammation, immune dysfunction, and long-term organ damage [2]. Patients with SLE now have improved survival rates due to advancements in diagnosis and therapy; however, as life expectancy increases, the risk of long-term metabolic and cardiovascular complications also rises, becoming a major clinical concern [3,4].

Metabolic syndrome (MetS) is a cluster of interconnected metabolic abnormalities, including central obesity, insulin resistance, dyslipidemia, and hypertension, which collectively increase the risk of type 2 diabetes mellitus (T2DM) and cardiovascular disease [5]. Patients with SLE are significantly more likely to develop MetS than the general population [6]. This increased susceptibility is considered multifactorial and may be attributed to chronic systemic inflammation, disease activity, physical inactivity, and prolonged exposure to corticosteroids and other immunosuppressive medications. These factors contribute to metabolic disturbances that may be overlooked in routine clinical care [7,8].

Moreover, the development of MetS in patients with SLE adds an additional burden to disease management and is associated with poorer clinical outcomes, including accelerated atherosclerosis and increased cardiovascular mortality [9]. Although this association is becoming increasingly recognized, reported prevalence rates vary across populations, possibly due to differences in genetic background, lifestyle factors, disease severity, and treatment regimens [10]. Furthermore, the specific factors associated with MetS development in patients with SLE remain poorly understood, particularly in South Asian populations, where baseline cardiovascular risk is high [11].

The prevalence of MetS among patients with SLE has been reported to range from approximately 3% to 45%, depending on the population studied and the diagnostic criteria used [12]. The development of MetS in SLE has been associated with several factors, including advanced age, prolonged disease duration, high disease activity, obesity, physical inactivity, lupus nephritis, and high corticosteroid doses [13]. Chronic inflammation and immune-mediated endothelial dysfunction also contribute to insulin resistance and the early development of atherosclerosis, thereby increasing cardiovascular risk in this population [14].

Although data regarding the prevalence of and factors associated with MetS among Pakistani patients with SLE are limited, emerging local evidence, together with international studies, suggests a growing burden of MetS in this population. Identifying modifiable and non-modifiable factors associated with MetS may help clinicians recognize patients at increased cardiometabolic risk and guide preventive and management strategies, particularly in South Asian populations where baseline cardiovascular risk is high. Therefore, this study was conducted to determine the prevalence of MetS and identify associated factors among patients with SLE attending a tertiary care hospital in Pakistan.

## Materials and methods

Study design

This hospital-based cross-sectional study aimed to determine the prevalence of MetS and identify its associated factors among patients with SLE.

Study setting

This study was conducted in the Department of Internal Medicine and Rheumatology at Khyber Teaching Hospital, Peshawar, Pakistan.

Study duration

This study was conducted over a two-year period from January 2024 to December 2025.

Study population

**Figure 1:**
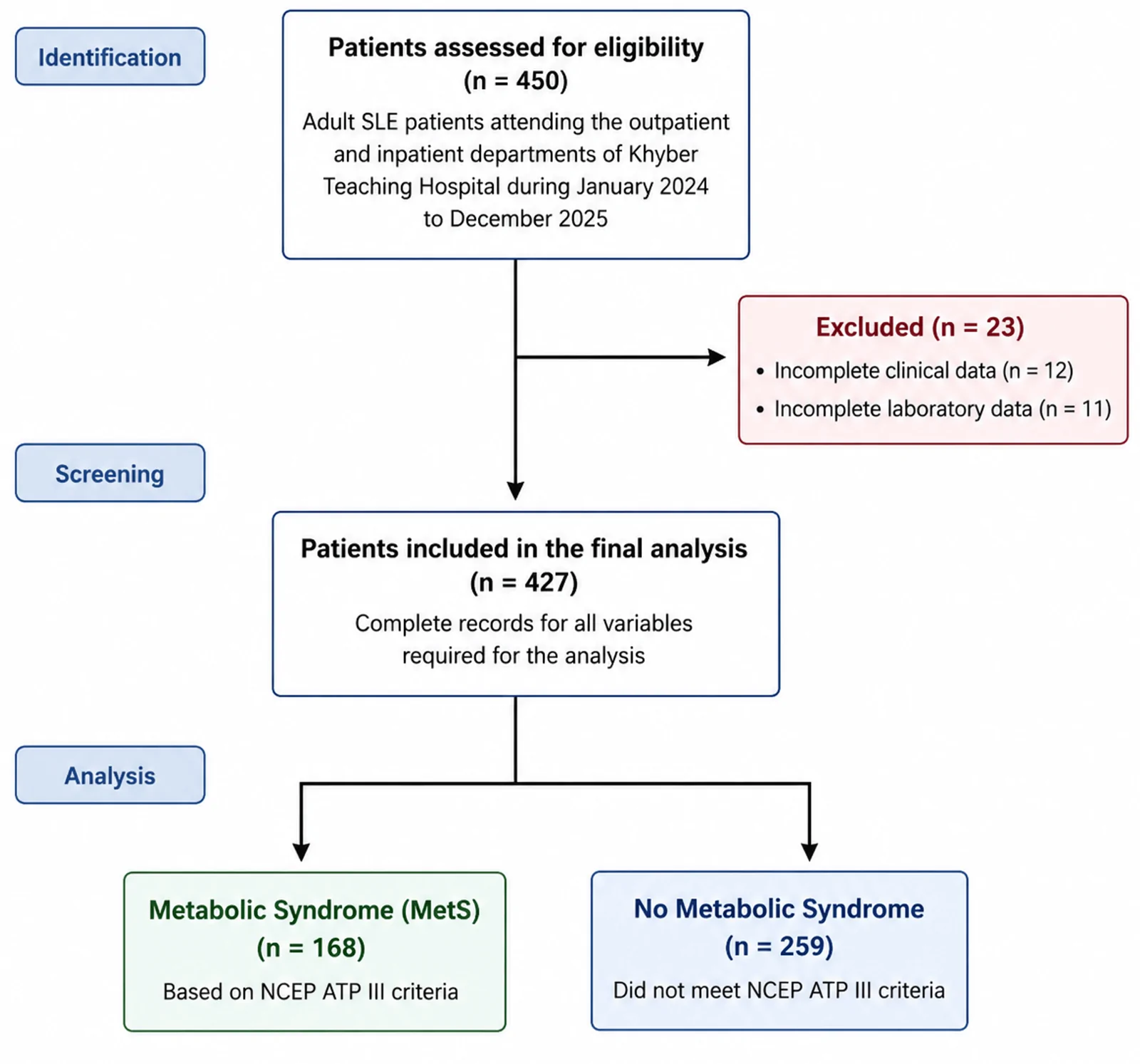
STROBE flow diagram of participant selection STROBE: Strengthening the Reporting of Observational Studies in Epidemiology.

All adult patients with SLE attending the outpatient and inpatient departments of Khyber Teaching Hospital during the study period were eligible for inclusion. Participants fulfilled the predefined SLE classification criteria, and demographic, clinical, laboratory, and treatment-related information were collected through medical record review and a structured data collection proforma. Information on lupus nephritis was obtained from documented clinical diagnoses recorded in the patients’ medical records by the treating rheumatologist and/or nephrologist. Participant recruitment and selection are illustrated in the Strengthening the Reporting of Observational Studies in Epidemiology (STROBE) flow diagram (Figure 1).

Sample size calculation

The sample size was calculated using the single population proportion formula:

$n=\frac{Z^{2}\times p(1-p)}{d^{2}}$

Here, p represents the anticipated prevalence of MetS among patients with SLE, d represents the margin of error, n represents the required sample size, and 1.96 represents the standard normal variate at a 95% confidence level. Because published estimates varied, the sample size was calculated using a reported prevalence of 50% to obtain the maximum sample size with a 5% margin of error and a 95% confidence level.

$n=\frac{1.96^{2}\times 0.50\times \left( 1-0.50 \right)}{0.50^{2}}$

$n=\frac{3.8416 &times; 0.25}{0.0025}$

*n=384.16*

The minimum required sample size of 384 was calculated using the single population proportion formula. An additional 10% was added, increasing the target sample size to 427, to account for non-response and incomplete data. During the study period, a total of 450 patients with SLE were approached and enrolled. However, 23 patients were excluded due to incomplete clinical or laboratory information. Therefore, 427 patients were included in the final analysis, as they provided complete data.

Sampling technique

A consecutive non-probability sampling technique was employed. All eligible adult patients with SLE attending the Rheumatology and Internal Medicine outpatient and inpatient departments during the study period were invited to participate consecutively until the required sample size was achieved. Consecutive recruitment of all eligible patients presenting during routine clinical services was performed to minimize, rather than eliminate, selection bias by reducing investigator discretion in participant selection and ensuring recruitment based on predefined eligibility criteria. Recruiting participants from both outpatient and inpatient settings throughout the study period enhanced the representativeness of the hospital-based study population. However, as this was a single-center study using a non-probability sampling strategy, the possibility of selection bias cannot be completely excluded, and the findings should be interpreted accordingly.

Inclusion and exclusion criteria

Patients were eligible for inclusion if they were aged 18 years or older, of either sex, and had a confirmed diagnosis of SLE based on the 2019 European League Against Rheumatism/American College of Rheumatology (EULAR/ACR) classification criteria [15]. Patients with incomplete clinical or laboratory data, pregnant women, and those with overlap autoimmune syndromes were excluded from the study.

Data collection procedure

Following written informed consent, demographic, clinical, laboratory, and treatment-related data were collected using a structured data collection proforma developed by the principal investigator and the research team in accordance with the study objectives (Appendix 1). The proforma was reviewed for content validity by senior faculty members from the Departments of Rheumatology and Internal Medicine at Khyber Teaching Hospital, Peshawar, and was pilot-tested in 20 patients with SLE to assess its clarity, feasibility, and completeness. Minor modifications were made following the pilot testing before commencement of the main study.

Data collected included age, sex, disease duration, lupus nephritis, smoking status, family history of diabetes mellitus, medication use (corticosteroids, hydroxychloroquine, and immunosuppressive agents), and physical activity. Physical activity was assessed by self-report and categorized as sedentary (<150 minutes of moderate-intensity physical activity per week) or active (≥150 minutes per week), consistent with the World Health Organization physical activity recommendations. Medication-related data included current use of corticosteroids, hydroxychloroquine, and immunosuppressive agents. Current corticosteroid use was defined as the use of systemic corticosteroid therapy at the time of study enrolment, as documented in the patient's medical record and current prescription. For participants receiving corticosteroids, the prescribed daily dose (prednisolone equivalent) at enrolment was recorded and categorized as low-, moderate-, or high-dose therapy.

Anthropometric measurements, including weight, height, body mass index (BMI), and waist circumference, were obtained using standardized procedures during the clinical examination. Body weight was measured to the nearest 0.1 kg using a calibrated digital weighing scale, whereas height was measured to the nearest 0.1 cm using a stadiometer, with participants wearing light clothing and no shoes. BMI was calculated as weight (kg) divided by height squared (m²). Waist circumference was measured at the midpoint between the lower margin of the last palpable rib and the iliac crest using a non-stretchable measuring tape, with the participant standing upright. Blood pressure was measured twice after at least five minutes of rest in the seated position using a calibrated sphygmomanometer, and the average of the two readings was recorded. Following an overnight fast of at least eight hours, venous blood samples were collected and analyzed in the hospital's central laboratory using standardized automated analyzers operating under internal and external quality-control procedures. Laboratory parameters included fasting plasma glucose, triglycerides, and high-density lipoprotein (HDL) cholesterol.

MetS was defined according to the National Cholesterol Education Program Adult Treatment Panel III (NCEP ATP III) diagnostic criteria. The diagnostic thresholds applied in this study were verified using the Endotext summary of MetS diagnostic criteria and the World Health Organization Eastern Mediterranean Regional Office (WHO EMRO) comparative table of MetS diagnostic guidelines [16,17]. Participants were classified as having MetS if they fulfilled three or more of the following five criteria: (1) waist circumference ≥102 cm in men or ≥88 cm in women; (2) serum triglycerides ≥150 mg/dL; (3) HDL cholesterol <40 mg/dL in men or <50 mg/dL in women; (4) blood pressure ≥130/85 mmHg or current use of antihypertensive medication; and (5) fasting plasma glucose ≥110 mg/dL. The predefined NCEP ATP III criteria were applied uniformly to all participants to ensure methodological consistency throughout data collection and analysis.

Study variables

The primary outcome variable was the presence of MetS, defined according to the NCEP ATP III diagnostic criteria. The candidate independent variables included demographic characteristics (age and sex), anthropometric measures (BMI), disease-related characteristics (disease duration and presence of lupus nephritis), lifestyle factors (smoking status and physical activity level), family history of diabetes mellitus, and treatment-related variables, including current systemic corticosteroid use, corticosteroid dose category, hydroxychloroquine use, and immunosuppressive therapy. These variables were selected a priori based on their biological plausibility, clinical relevance, and previously reported associations with MetS among patients with systemic lupus erythematosus. To avoid multicollinearity and over-adjustment, variables constituting the diagnostic components of MetS (waist circumference, blood pressure, fasting plasma glucose, triglycerides, and HDL cholesterol) were excluded from the multivariable logistic regression model. Although BMI is correlated with waist circumference, it was retained because it reflects general adiposity rather than central obesity and has been independently associated with metabolic abnormalities in patients with SLE.

Data analysis

Data were analyzed using IBM SPSS Statistics for Windows, Version 25.0 (Released Year; IBM Corp., Armonk, NY, USA). Categorical variables were summarized as frequencies and percentages, whereas continuous variables were expressed as mean ± standard deviation (SD). The normality of continuous variables was assessed before selecting the appropriate statistical tests. The prevalence of MetS was calculated as the proportion of participants who fulfilled the NCEP ATP III diagnostic criteria.

Comparisons between participants with and without MetS were performed using the chi-square test (or Fisher's exact test, where appropriate) for categorical variables and the independent-samples t-test for continuous variables. Candidate variables eligible for multivariable analysis included age, sex, BMI, disease duration, lupus nephritis, smoking status, physical activity level, family history of diabetes mellitus, corticosteroid use, corticosteroid dose category, hydroxychloroquine use, and immunosuppressive therapy. Variables demonstrating an association with MetS at p < 0.20 in the univariate analysis, together with clinically relevant variables identified a priori based on existing evidence, were entered simultaneously into a multivariable logistic regression model using the Enter method to identify factors independently associated with MetS.

Variables constituting the diagnostic components of MetS were intentionally excluded from the regression model to avoid multicollinearity and over-adjustment, as these variables formed part of the outcome definition. Model results are presented as adjusted odds ratios (AORs) with 95% confidence intervals (CIs). A complete-case analysis was performed, and participants with incomplete clinical or laboratory data were excluded from the final analysis. Of the 450 participants initially assessed for eligibility, 23 (5.1%) were excluded because of incomplete data, leaving 427 participants in the final analysis. Model adequacy was assessed using the Hosmer-Lemeshow goodness-of-fit test, and multicollinearity among the independent variables was evaluated before fitting the final regression model. A two-tailed p-value < 0.05 was considered statistically significant.

Ethical considerations

The study received ethical approval from the Institutional Research and Ethics Board (IREB) of Khyber Medical College (KMC)/Khyber Teaching Hospital (KTH), Peshawar, before the commencement of the study (Approval No. 973/IM/KMC; dated 12 December 2023). Written informed consent was obtained from all participants before enrolment. All data collected were treated confidentially and anonymously and were used solely for research purposes.

## Results

The study cohort comprised predominantly young-to-middle-aged women, with a substantial burden of overweight and obesity affecting nearly 6 in 10 patients. The majority had been living with SLE for more than five years, and corticosteroid exposure was nearly universal. These baseline characteristics describe a population already at high metabolic risk due to female sex, adiposity, chronic glucocorticoid exposure, and prolonged disease duration (Table 1).

**Table 1:** Demographic, lifestyle, clinical, and treatment characteristics of patients with systemic lupus erythematosus (n = 427) ^†^Corticosteroid dose categories: low (<7.5 mg/day prednisone equivalent), moderate (7.5-30 mg/day prednisone equivalent), and high (>30 mg/day prednisone equivalent). *Sedentary lifestyle: <150 minutes/week of moderate-intensity physical activity.

Variable	Category	Frequency (%)
Age (years)	18-30	138 (32.32)
	31-45	196 (45.91)
	>45	93 (21.78)
Sex	Male	52 (12.18)
	Female	375 (87.82)
BMI category	Normal	171 (40.05)
	Overweight	158 (37.02)
	Obese	98 (22.95)
Smoking status	Yes	74 (17.34)
	No	353 (82.66)
Physical activity	Sedentary*	241 (56.44)
	Active	186 (43.56)
Disease duration	≤5 years	189 (44.27)
	>5 years	238 (55.73)
Lupus nephritis	Yes	162 (37.94)
	No	265 (62.06)
Current corticosteroid use	Yes	356 (83.37)
	No	71 (16.63)
Corticosteroid dose^†^	Low	158 (44.38)
	Moderate	132 (37.08)
	High	66 (18.54)
Hydroxychloroquine use	Yes	289 (67.68)
	No	138 (32.32)
Immunosuppressive therapy	Yes	221 (51.76)
	No	206 (48.24)
Family history of diabetes mellitus	Yes	146 (34.19)
	No	281 (65.81)

Biochemical profiling demonstrated an adverse cardiometabolic risk profile in the study population. Mean BMI and waist circumference indicated central adiposity, while blood pressure, fasting plasma glucose, and triglyceride levels were close to or exceeded conventional diagnostic thresholds. The low mean HDL cholesterol level further reflected an atherogenic lipid profile, suggesting the presence of metabolic abnormalities at the time of assessment (Table 2).

**Table 2:** Anthropometric, clinical, laboratory, and disease characteristics of patients with systemic lupus erythematosus (n = 427)

Variable	Mean ± SD
BMI (kg/m²)	27.80 ± 5.12
Waist circumference (cm)	91.48 ± 12.36
Systolic blood pressure (mmHg)	128.62 ± 14.91
Diastolic blood pressure (mmHg)	82.44 ± 9.87
Fasting plasma glucose (mg/dL)	102.35 ± 18.72
Triglycerides (mg/dL)	162.84 ± 48.39
High-density lipoprotein cholesterol (mg/dL)	43.27 ± 10.11
SLEDAI-2K score	6.8 ± 4.2
SLICC/ACR Damage Index (SDI)	1.1 ± 1.4

Univariate analysis identified several factors associated with MetS. Obesity showed the strongest crude association, followed by corticosteroid use and physical inactivity. Disease-related factors, including lupus nephritis and longer disease duration, were also significantly associated with MetS, as was a family history of diabetes mellitus. Smoking status and hydroxychloroquine use showed borderline statistical significance and met the prespecified criterion for inclusion in the multivariable logistic regression analysis (Table 3).

**Table 3:** Univariate logistic regression analysis of factors associated with metabolic syndrome among patients with systemic lupus erythematosus

Variable	χ²	Crude OR (95% CI)	p-value
Age > 45 years	18.42	2.89 (1.88-4.45)	<0.001
BMI ≥ 30 kg/m²	34.11	4.12 (2.65-6.39)	<0.001
Disease duration > 5 years	10.76	2.01 (1.34-3.01)	0.001
Lupus nephritis	8.94	1.78 (1.20-2.65)	0.004
SLEDAI-2K score (per 1 point)	12.48	1.12 (1.05-1.20)	0.001
SLICC/ACR SDI score (per 1 point)	14.15	1.35 (1.15-1.58)	<0.001
Smoking	3.62	1.69 (0.98-2.91)	0.060
Sedentary lifestyle	25.37	2.91 (1.92-4.41)	<0.001
Family history of diabetes mellitus	8.15	1.84 (1.21-2.79)	0.004
Current corticosteroid use	28.96	3.22 (1.86-5.57)	<0.001
Hydroxychloroquine use	3.41	0.69 (0.47-1.02)	0.060
Immunosuppressive therapy	3.08	1.38 (0.96-1.98)	0.083

The MetS group was significantly older, had higher BMI and waist circumference, and exhibited significantly higher blood pressure, fasting plasma glucose, and triglyceride levels, along with significantly lower HDL cholesterol levels, than the group without MetS. All continuous variables differed significantly between the two groups, indicating distinct anthropometric and biochemical differences between participants with and without MetS (Table 4).

**Table 4:** Comparison of anthropometric, clinical, laboratory, and disease characteristics between participants with and without metabolic syndrome

Variable	MetS (n = 168)	Non-MetS (n = 259)	Test statistic	p-value
Age (years)	44.62 ± 10.84	36.91 ± 11.27	7.21	<0.001
BMI (kg/m²)	31.28 ± 5.12	25.67 ± 4.89	11.34	<0.001
Waist circumference (cm)	101.45 ± 9.82	85.36 ± 10.44	15.02	<0.001
Systolic BP (mmHg)	136.72 ± 12.94	123.18 ± 13.61	10.28	<0.001
Diastolic BP (mmHg)	88.64 ± 8.92	79.21 ± 9.04	10.89	<0.001
Fasting glucose (mg/dL)	118.56 ± 16.22	91.72 ± 14.88	17.45	<0.001
Triglycerides (mg/dL)	198.41 ± 52.67	138.92 ± 41.33	12.88	<0.001
HDL cholesterol (mg/dL)	37.84 ± 8.92	46.12 ± 9.33	-9.41	<0.001
SLEDAI-2K score	8.2 ± 4.5	5.9 ± 3.8	5.42	<0.001
SLICC/ACR SDI score	1.6 ± 1.5	0.8 ± 1.2	5.85	<0.001

After adjustment for potential confounders, obesity remained the strongest independent predictor of MetS, whereas physical inactivity and corticosteroid use were each associated with approximately threefold higher odds of MetS. Older age, longer disease duration, lupus nephritis, and a family history of diabetes mellitus also remained independently associated with MetS. Hydroxychloroquine use was independently associated with lower odds of MetS. In contrast, smoking was not independently associated with MetS after multivariable adjustment (Table 5).

**Table 5:** Multivariable logistic regression analysis of factors independently associated with metabolic syndrome among patients with systemic lupus erythematosus Model diagnostics: Omnibus test of model coefficients: χ² = 156.42, df = 12, p < 0.001; Hosmer-Lemeshow goodness-of-fit test: p = 0.38; Nagelkerke R² = 0.43. Variables that constitute the diagnostic components of metabolic syndrome (waist circumference, blood pressure, fasting plasma glucose, triglycerides, and HDL cholesterol) were excluded from the multivariable model to avoid multicollinearity and over-adjustment, as they are part of the outcome definition.

Variable	Adjusted OR	95% CI	Wald χ²	p-value
Age > 45 years	2.41	1.52-3.82	14.62	<0.001
BMI ≥ 30 kg/m²	3.76	2.34-6.04	28.91	<0.001
Disease duration > 5 years	1.89	1.21-2.96	7.89	0.005
Lupus nephritis	1.67	1.08-2.59	5.32	0.021
SLEDAI-2K score (per 1-point)	1.06	0.98-1.15	2.76	0.097
SLICC/ACR SDI score (per 1-point)	1.28	1.08-1.52	6.52	0.011
Smoking	1.54	0.88-2.70	2.29	0.130
Sedentary lifestyle	2.58	1.66-4.01	16.84	<0.001
Family history of diabetes mellitus	1.72	1.13-2.62	6.40	0.011
Current corticosteroid use	2.96	1.55-5.64	10.91	0.001
Hydroxychloroquine use	0.64	0.43-0.95	4.84	0.028
Immunosuppressive therapy	1.15	0.76-1.74	0.48	0.490

Disease activity was distributed across the spectrum, with most patients exhibiting mild-to-moderate disease activity and a smaller but clinically important proportion exhibiting high or very high disease activity. Cumulative organ damage was present in more than half of the cohort, with approximately one in nine patients demonstrating substantial organ damage. These findings indicate that the study population included patients with varying levels of disease activity and cumulative organ damage (Table 6).

**Table 6:** Disease activity and organ damage categories among patients with systemic lupus erythematosus (n = 427)

Variable	Category	Frequency (%)
SLEDAI-2K	No activity (0)	78 (18.27)
	Mild (1-5)	137 (32.08)
	Moderate (6-10)	119 (27.87)
	High (11-19)	73 (17.10)
	Very high (≥20)	20 (4.68)
SLICC/ACR SDI	0	192 (44.96)
	1	120 (28.10)
	2	65 (15.22)
	≥3	50 (11.71)

## Discussion

In the present study, MetS was identified in 168 of 427 patients with SLE, yielding a prevalence of 39.34% (95% CI, approximately 34.7%-44.0%). MetS was defined using the NCEP ATP III diagnostic criteria, whereby participants were classified as having MetS if they fulfilled at least three of the following five criteria: waist circumference ≥102 cm in men or ≥88 cm in women, serum triglycerides ≥150 mg/dL, HDL cholesterol <40 mg/dL in men or <50 mg/dL in women, blood pressure ≥130/85 mmHg (or current antihypertensive therapy), and fasting plasma glucose ≥110 mg/dL. The prevalence observed in our study falls within the range reported internationally (16%-45%) and closely resembles findings from large SLE cohorts reporting prevalence estimates of approximately 35%-38% using comparable diagnostic criteria [18]. The relatively high prevalence observed in our cohort may reflect the combined effects of chronic systemic inflammation, prolonged corticosteroid exposure, and the elevated background cardiometabolic risk characteristic of South Asian populations.

The demographic and clinical profile of our cohort provides important context for interpreting these findings. The study population was predominantly female (87.82%), consistent with the known epidemiology of SLE, and the majority of participants were between 31 and 45 years of age [19]. Furthermore, participants were recruited consecutively from both outpatient clinics and inpatient wards of a tertiary care hospital, enabling the inclusion of patients with varying degrees of disease severity and enhancing the representativeness of the hospital-based study population. Nevertheless, the prevalence estimates should be interpreted within the context of this tertiary care setting and may not be directly generalizable to community-based SLE populations.

The metabolic characteristics of our patients further emphasized their increased cardiometabolic vulnerability. The mean BMI was 27.80 ± 5.12 kg/m², the mean waist circumference was 91.48 ± 12.36 cm, and the mean fasting plasma glucose level was 102.35 ± 18.72 mg/dL, suggesting a high burden of adiposity and early disturbances in glucose metabolism. The most prevalent individual components of MetS were low HDL cholesterol (51.76%), central obesity (50.12%), and hypertension (45.20%), followed by elevated triglycerides and fasting plasma glucose. This pattern is consistent with previous studies demonstrating that dyslipidemia, particularly reduced HDL cholesterol, together with central obesity, represents the predominant metabolic abnormality among patients with SLE [20]. These findings support the concept that chronic inflammation, altered lipid metabolism, corticosteroid therapy, and reduced physical activity collectively contribute to metabolic dysfunction in this patient population.

Univariate analysis demonstrated significant associations between MetS and older age, obesity, longer disease duration, lupus nephritis, physical inactivity, a family history of diabetes mellitus, and corticosteroid use. These findings are consistent with previous studies reporting that these clinical and lifestyle characteristics are commonly associated with metabolic abnormalities among patients with SLE [21]. Corticosteroid exposure was particularly common in our cohort, with 83.37% of patients receiving systemic corticosteroids, including low-, moderate-, and high-dose regimens. Although glucocorticoid therapy has recognized metabolic effects, current corticosteroid use may also reflect greater disease activity or severity. Consequently, the observed association should be interpreted as an association rather than evidence of a causal relationship.

After adjustment for potential confounding variables, obesity (BMI ≥ 30 kg/m²), corticosteroid use, physical inactivity, older age, longer disease duration, lupus nephritis, and a family history of diabetes mellitus remained independently associated with higher odds of MetS, whereas hydroxychloroquine use was independently associated with lower odds of MetS. This finding is consistent with previous reports demonstrating favorable effects of hydroxychloroquine on glucose homeostasis and lipid metabolism in patients with SLE [22]. However, because both the exposure variables and MetS status were measured simultaneously, the temporal sequence of these associations cannot be determined. For example, physical inactivity may contribute to the development of metabolic abnormalities, but patients with obesity, active SLE, lupus nephritis, MetS, or disease-related physical limitations may also engage in less physical activity. Similarly, corticosteroid use may reflect underlying disease severity rather than being solely responsible for the observed metabolic abnormalities. Therefore, these findings should be interpreted as independent associations rather than causal effects, and prospective longitudinal studies are warranted to clarify the temporal relationships and causality of these associations.

The observed association between lupus nephritis and MetS further supports previous evidence linking renal involvement with accelerated cardiovascular risk in SLE through persistent systemic inflammation, proteinuria-associated dyslipidemia, and the need for more intensive immunosuppressive and corticosteroid therapy [18-20]. Overall, our findings indicate that the occurrence of MetS in patients with SLE reflects a complex interaction among disease-related characteristics, treatment exposures, and modifiable lifestyle factors. These findings highlight the importance of routine metabolic risk assessment, optimization of corticosteroid therapy whenever clinically feasible, promotion of regular physical activity and weight management, and continued use of hydroxychloroquine, where appropriate, to reduce long-term cardiometabolic risk in patients with SLE.

Strengths and limitations

This study has several important strengths that enhance the validity and clinical relevance of its findings. First, the relatively large sample size improved the precision of the prevalence estimate and provided adequate statistical power to evaluate factors independently associated with MetS. Second, MetS was defined using the NCEP ATP III diagnostic criteria, facilitating comparison of the findings with previous international studies that used the same definition. Third, comprehensive demographic, clinical, anthropometric, biochemical, lifestyle, treatment-related, and disease-specific data were collected using a standardized data collection proforma, enabling a broad evaluation of factors associated with MetS among patients with SLE. Finally, multivariable logistic regression analysis was performed to adjust for potential confounding variables while avoiding over-adjustment by excluding individual diagnostic components of MetS from the regression model. Because BMI reflects overall adiposity rather than central obesity, an additional sensitivity analysis excluding BMI from the multivariable model was performed. The overall pattern of associations remained materially unchanged, supporting the robustness of the study findings.

Several limitations should also be considered when interpreting these findings. First, the cross-sectional design precludes establishing temporal relationships or causal inferences between the examined variables and MetS; therefore, the observed associations should not be interpreted as causal. Second, although BMI was included in the primary multivariable model as an indicator of general adiposity, it is not a diagnostic component of the NCEP ATP III definition of MetS. Waist circumference remains the preferred measure of central obesity; therefore, the association observed for BMI should be interpreted cautiously. Reassuringly, the sensitivity analysis excluding BMI yielded similar findings, indicating that the principal associations identified in this study were robust. Third, the use of consecutive non-probability sampling in a single tertiary care hospital may have introduced selection bias and limits the generalizability of the findings to the broader SLE population. Although consecutive recruitment of all eligible patients was undertaken to minimize selection bias, it could not eliminate this possibility completely. Fourth, residual confounding from unmeasured variables, such as dietary habits, socioeconomic status, cumulative corticosteroid exposure, disease activity, and physical fitness, cannot be excluded. Finally, although this study was conducted in a South Asian population, the use of NCEP ATP III waist circumference thresholds, which are higher than the ethnic-specific cut-offs recommended for South Asian populations, may have resulted in underestimation of central obesity and, consequently, MetS prevalence. Future multicenter prospective studies using ethnic-specific diagnostic thresholds are warranted to validate and extend these findings.

## Conclusions

This study demonstrates that MetS is common among patients with SLE and is independently associated with a combination of demographic, disease-related, treatment-related, and lifestyle factors. These findings highlight the importance of incorporating routine metabolic screening and comprehensive assessment of metabolic abnormalities into the clinical management of patients with SLE. Early identification of MetS, along with appropriate lifestyle interventions and optimization of disease management, may improve overall metabolic health in this population. Given the cross-sectional nature of the study, the observed associations should not be interpreted as causal, and prospective multicenter longitudinal studies are warranted to clarify temporal relationships, evaluate disease progression, and validate these findings.

## Appendices

Appendix 1 

**Table 7:** Prevalence and predictors of metabolic syndrome in patients with systemic lupus erythematosus (SLE)

Section	Variable	Response options/units
Study information	Study ID	__________
	Date of enrolment	____ / ____ / ______
A. Sociodemographic characteristics	Age	______ years
	Sex	☐ Male ☐ Female
	Marital status	☐ Single ☐ Married ☐ Divorced/Widowed
	Education level	☐ Illiterate ☐ Primary ☐ Secondary ☐ Graduate ☐ Postgraduate
	Occupation	__________________
	Residence	☐ Urban ☐ Rural
B. Lifestyle factors	Smoking status	☐ Current smoker ☐ Former smoker ☐ Never smoker
	Physical activity	☐ Sedentary (<150 minutes/week of moderate-intensity physical activity) ☐ Active (≥150 minutes/week)
	Dietary pattern (optional)	☐ Balanced ☐ High-fat ☐ High-carbohydrate ☐ Mixed
C. Clinical characteristics of SLE	Duration of SLE	______ years
	Disease duration category	☐ ≤5 years ☐ >5 years
	Lupus nephritis	☐ Yes ☐ No
	Basis of lupus nephritis diagnosis	☐ Renal biopsy ☐ Clinical/laboratory diagnosis documented by treating physician ☐ Both
	SLE Disease Activity Index (SLEDAI-2K)	Score: ______
	Disease activity category	☐ Remission ☐ Mild ☐ Moderate ☐ High ☐ Very high
	SLICC/ACR Damage Index (SDI)	Score: ______
D. Medication history	Current systemic corticosteroid use (at enrolment)	☐ Yes ☐ No
	Duration of corticosteroid therapy	______ months/years
	Current daily dose (prednisolone equivalent)	______ mg/day
	Dose category	☐ Low (<7.5 mg/day) ☐ Moderate (7.5-30 mg/day) ☐ High (>30 mg/day)
	Hydroxychloroquine use	☐ Yes ☐ No
	Immunosuppressive therapy	☐ Yes ☐ No
	If yes, specify	__________________
E. Family history	Diabetes mellitus	☐ Yes ☐ No
	Hypertension	☐ Yes ☐ No
	Cardiovascular disease	☐ Yes ☐ No
F. Anthropometric measurements	Weight	______ kg
	Height	______ cm
	Body mass index (BMI)	______ kg/m²
	Waist circumference	______ cm
G. Blood pressure	Systolic blood pressure	______ mmHg
	Diastolic blood pressure	______ mmHg
H. Laboratory investigations	Fasting plasma glucose	______ mg/dL
	Triglycerides	______ mg/dL
	HDL cholesterol	______ mg/dL
I. Metabolic syndrome assessment (NCEP ATP III)	Central obesity (waist circumference criterion)	☐ Yes ☐ No
	Elevated triglycerides (≥150 mg/dL)	☐ Yes ☐ No
	Reduced HDL cholesterol (<40 mg/dL in men; <50 mg/dL in women)	☐ Yes ☐ No
	Elevated blood pressure (≥130/85 mmHg or antihypertensive treatment)	☐ Yes ☐ No
	Elevated fasting plasma glucose (≥110 mg/dL)	☐ Yes ☐ No
	Number of MetS criteria fulfilled	0 ☐ 1 ☐ 2 ☐ 3 ☐ 4 ☐ 5
	Metabolic syndrome present	☐ Yes (≥3 criteria) ☐ No (<3 criteria)
